# Development and validation of a prediction model for hypoproteinemia after traumatic spinal cord injury: A multicenter retrospective clinical study

**DOI:** 10.1097/MD.0000000000038081

**Published:** 2024-06-21

**Authors:** Xiuwei Tan, Yanlan Wu, Fengxin Li, Qian Wei, Xuefeng Lu, Xiaoxi Huang, Deshen He, Xiaozhen Huang, Shiquan Deng, Linting Hu, Fangming Song, Yiji Su

**Affiliations:** a The First Affiliated Hospital of Guangxi Medical University, Nanning, China; b The People’s Hospital of Dahua Yao Autonomus County, Hechi, China; c Wuzhou GongRen Hospital, Wuzhou, China; d Guangxi Health Science College, Nanning, China; e Guangxi Medical University, Nanning, China.

**Keywords:** hypoproteinemia, nomogram, traumatic spinal cord injury

## Abstract

A multicenter retrospective analysis of conventionally collected data. To identify the potential causes of hypoproteinemia after traumatic spinal cord injury (TSCI) and provide a diagnostic model for predicting an individual likelihood of developing hypoproteinemia. Hypoproteinemia is a complication of spinal cord injury (SCI), an independent risk factor for respiratory failure in elderly patients with SCI, and a predictor of outcomes in patients with cervical SCI. Few nomogram-based studies have used clinical indicators to predict the likelihood of hypoproteinemia following TSCI. This multicenter retrospective clinical analysis included patients with TSCI admitted to the First Affiliated Hospital of Guangxi Medical University, Wuzhou GongRen Hospital, and Dahua Yao Autonomous County People Hospital between 2016 and 2020. The data of patients from the First Affiliated Hospital of Guangxi Medical University were used as the training set, and those from the other 2 hospitals were used as the validation set. All patient histories, diagnostic procedures, and imaging findings were recorded. To predict whether patients with TSCI may develop hypoproteinemia, a least absolute shrinkage and selection operator regression analysis was conducted to create a nomogram. The model was validated by analyzing the consequences using decision curve analysis, calibration curves, the C-index, and receiver operating characteristic curves. After excluding patients with missing data, 534 patients were included in this study. Male/female sex, age ≥ 60 years, cervical SCI, pneumonia, pleural effusion, urinary tract infection (UTI), hyponatremia, fever, hypotension, and tracheostomy were identified as independent risk factors of hypoalbuminemia. A simple and easy-to-replicate clinical prediction nomogram was constructed using these factors. The area under the curve was 0.728 in the training set and 0.881 in the validation set. The predictive power of the nomogram was satisfactory. Hypoalbuminemia after TSCI may be predicted using the risk factors of male/female sex, age ≥ 60 years, cervical SCI, pneumonia, pleural effusion, UTI, hyponatremia, fever, hypotension, and tracheostomy.

## 1. Introduction

Traumatic spinal cord injury (TSCI) is defined as damage to the structure and function of the spinal cord caused by trauma.^[1]^ With the aging of the population and increased life expectancy, the incidence of TSCI is gradually increasing. This increase is more pronounced in China and Canada as the populations of these countries are aging rapidly.^[2,3]^ The mortality and disability rates associated with TSCI are high.^[4]^ TSCI can also lead to spinal cord dysfunction. Patients with TSCI often have tetraplegia, functional defects of the autonomic nervous system, and urination and defecation disorders, which cause physical and emotional difficulties to patients and place a huge burden on society and families.^[3,5]^ TSCI can also be fatal. Therefore, effectively managing TSCI and providing patients with better rehabilitation outcomes has become a topic that social and medical practitioners must address.

Hypoproteinemia, a complication of spinal cord injury (SCI), is an independent risk factor for respiratory failure in elderly patients with SCI^[6]^ and a predictor of outcomes in patients with cervical SCI.^[7]^ Pneumonia is one of the main causes of death in patients with SCI.^[8]^ In patients with stroke, hypoalbuminemia is an independent risk factor for pneumonia during the rehabilitation period.^[9]^ Low serum albumin is an important factor affecting medical success and the adequacy of a patient response to trauma and disease, life, and longevity. Spontaneous changes in serum albumin levels are an important index of health recovery or deterioration in critical diseases.^[10]^ Hypoproteinemia can be diagnosed using blood samples, though frequent blood draws are painful and costly. Therefore, hypoproteinemia is not widely used as a health indicator in patients with TSCI, especially in China, which is a developing country with an unbalanced regional economy. If hypoproteinemia prediction and screening could be performed during the early stages of TSCI, regular blood tests will only need to be conducted for high-risk patients to improve the detection accuracy, thus reducing the adverse reactions of blood tests and decreasing medical expenses.

Clinical prediction models are widely used for the clinical diagnosis of specific diseases and the prediction of clinical outcomes.^[11]^ In recent years, logistic regression, Cox regression, nomograms, least absolute shrinkage and selection operator (LASSO) regression, machine learning, and other methods have been widely applied.^[12,13]^ The nomogram prediction model has great application value in the diagnosis, treatment selection, and assessment of prognosis in several diseases, including prostate cancer,^[14]^ colorectal cancer,^[15]^ and SCI.^[16]^ It has positively affected the prediction of different diseases and conditions in clinical settings. However, few studies regarding predictive models of hypoproteinemia in patients with TSCI have been reported. Further, we have not retrieved the relevant studies on the risk of nomogram prediction model of hypoproteinemia after TSCI.

Therefore, there is an urgent need to develop early screening tools for post-TSCI hypoproteinemia. This study reports the development and verification of a predictive model for hypoproteinemia after TSCI. The model uses patients’ health information to achieve early screening and potential treatment for hypoproteinemia in patients with TSCI.

## 2. Methods

### 2.1. Study population

The clinical data of patients with TSCI hospitalized at the First Affiliated Hospital of Guangxi Medical University, Wuzhou GongRen Hospital, and Dahua Yao Autonomous County People Hospital from 2016 to 2020 were collected. Patients who met the diagnostic criteria for TSCI based on imaging and clinical data who had no missing data in their medical records were included in this study. Patients with spinal diseases and other chronic underlying diseases affecting spinal cord function prior to admission, congenital malformations, SCI caused by non-traumatic causes, or incomplete medical records were excluded from this study. This study was approved by the ethics committees of the First Affiliated Hospital of Guangxi Medical University, Wuzhou GongRen Hospital, and Dahua Yao Autonomous County People Hospital.

### 2.2. Data collection

The patients were divided into 2 groups based on the presence or absence of hypoproteinemia. Patient sex, age, medical history, diet, urination and defecation functions, blood pressure, injury segment, fever, deep vein ultrasound findings, routine blood test results, serum albumin level, blood gas analysis findings, complications, tracheotomy status, surgery, and imaging results were collected. Patients from the First Affiliated Hospital of Guangxi Medical University were used as the training set, while those from the other 2 hospitals were used as the validation set.

### 2.3. Statistical analysis

Statistical analyses were performed using R software (Version 4.3.1) and auxiliary RStudio software (Version 2023.6.0.421). Statistical significance was set at *P *< .05. Logistic regression was used for the preliminary data analysis, and a LASSO regression analysis was conducted to identify the factors that should be included in the prediction model. The nomogram model was assessed using the C-index. The actual and predicted risks of hypoalbuminemia after TSCI were evaluated using the calibration curves. The area under the curve was used to evaluate the predictive power of the nomogram, and the net benefit of the nomogram was quantified using a decision curve analysis to assess its clinical benefit. The nomogram was verified using an external verification method.

## 3. Results

A total of 550 patients with TSCI were enrolled between 2016 and 2020, though 16 patients were excluded due to incomplete data. Finally, 534 patients were included in this study. The patients’ general data, clinical manifestations, blood examination results, imaging findings, rehabilitation assessment results, and other clinical data are shown in Table 1.

**Table 1:** Baseline data analysis of patients with and without hypoproteinemia after TSCI.

Characteristics	Hypoproteinemia	Non-hypoproteinemia	*P* value
n	237	297	
Gender, n (%)			.531
Male	191 (35.8%)	218 (40.8%)	
Female	46 (8.6%)	79 (14.8%)	
Age			.004
≥ 60	72 (13.5%)	58 (10.9%)	
<60	165 (30.9%)	239 (44.8%)	
Segments, n (%)			.457
Cervical	145 (27.2%)	191 (35.8%)	
Non-cervical	92 (17.2%)	106 (19.9%)	
Pneumonia, n (%)			<.001
Yes	150 (28.1%)	98 (18.4%)	
No	87 (28.1%)	199 (37.3%)	
Pleural effusion, n (%)			<.001
Yes	144 (27%)	87 (16.3%)	
No	93 (17.4%)	210 (39.3%)	
UTI*, n (%)			.693
No	183 (34.3%)	225 (42.1%)	
Yes	54 (10.1%)	72 (13.5%)	
DVT†, n (%)			<.001
No	190 (35.6%)	275 (51.5%)	
Yes	47 (8.8%)	22 (4.1%)	
Fever, n (%)			<.001
No	75 (14%)	180 (33.7%)	
Yes	162 (30.3%)	117 (21.9%)	
Constipation, n (%)			.123
No	168 (31.5%)	228 (42.7%)	
Yes	69 (12.9%)	69 (12.9%)	
Intestinal obstruction, n (%)			.007
No	229 (42.9%)	296 (55.4%)	
Yes	8 (1.5%)	1 (0.2%)	
Hypoxemia, n (%)			<.001
No	203 (38%)	283 (53%)	
Yes	34 (6.4%)	14 (2.6%)	
Hypotension, n (%)			<.001
No	199 (37.3%)	283 (53%)	
Yes	38 (7%)	14 (2.6%)	
DM‡, n (%)			.099
No	224 (41.9%)	289 (54.1%)	
Yes	13 (2.4%)	8 (1.5%)	
Hypertension, n (%)			.446
No	209 (39.1%)	268 (50.2%)	
Yes	28 (5.2%)	29 (5.4%)	
Tracheostomy, n (%)			.005
No	213 (39.9%)	285 (53.4%)	
Yes	24 (4.5%)	12 (2.2%)	
Diarrhea, n (%)			.008
No	220 (41.2%)	290 (54.3%)	
Yes	17 (3.2%)	7 (1.3%)	
Operation, n (%)			.017
Yes	198 (37.1%)	223 (41.8%)	
No	39 (7.3%)	74 (13.9%)	

TSCI = traumatic spinal cord injury.

* UTI: urinary tract infection.

† DVT: deep vein thrombosis.

‡ DM: diabetes mellitus.

**Figure 1.**
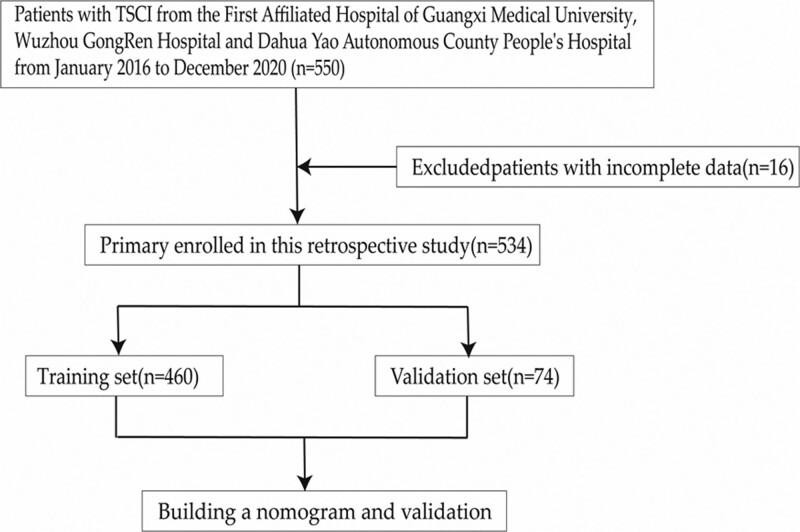
Flow chart for selection of hypoproteinemia after TSCI. TSCI = traumatic spinal cord injury.

**Figure 2.**
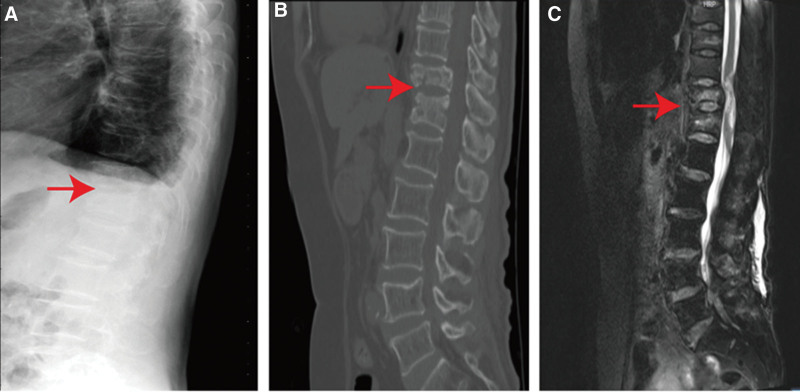
Radiographic examination of the patient with TSCI. (A) Lateral radiographs, the arrow points to the 11 to 12 thoracic fractures. (B) Computed tomography (CT) in the sagittal position, the arrow points to the 11 to 12 thoracic fractures. (C) Magnetic resonance imaging (MRI) in the sagittal position, the arrow points to injured thoracic spinal cord. TSCI = traumatic spinal cord injury.

**Figure 3.**
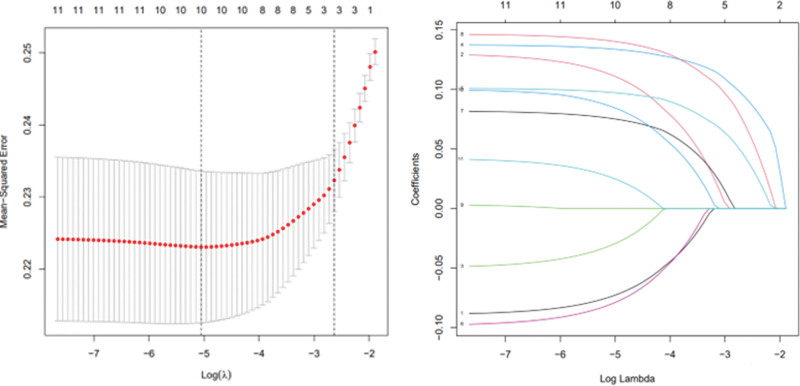
All perioperative parameters were calculated in lasso analysis. (A) Binomial deviance was plotted using the lasso binary logistic regression model, and 10 parameters were statistically significant. (B) Coefficient profiles of the 10 features were plotted using the lasso binary logistic regression model. LASSO = least absolute shrinkage and selection operator.

**Figure 4.**
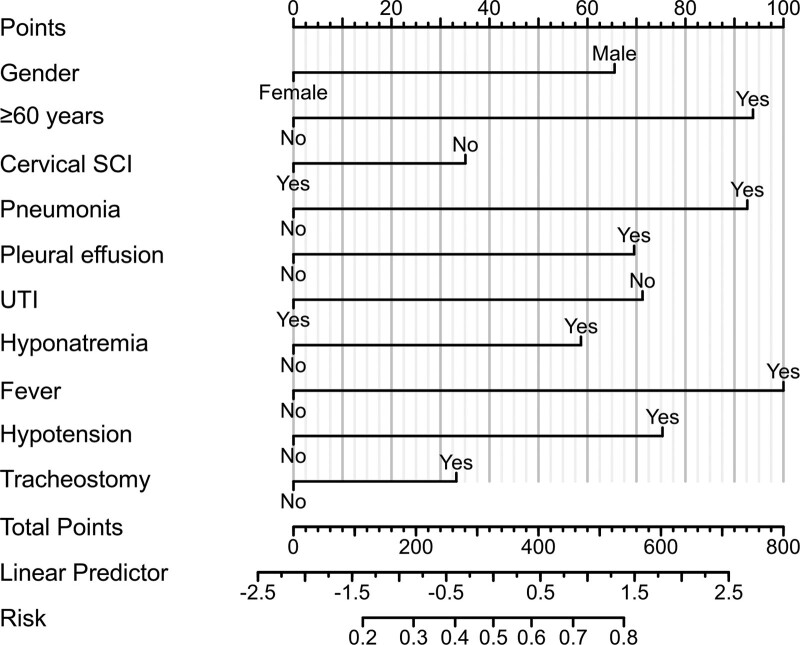
A novel nomogram was constructed to predict hypoproteinemia after TSCI risk by calculating the total score of 10 parameters. TSCI = traumatic spinal cord injury, UTI = urinary tract infection.

**Figure 5.**
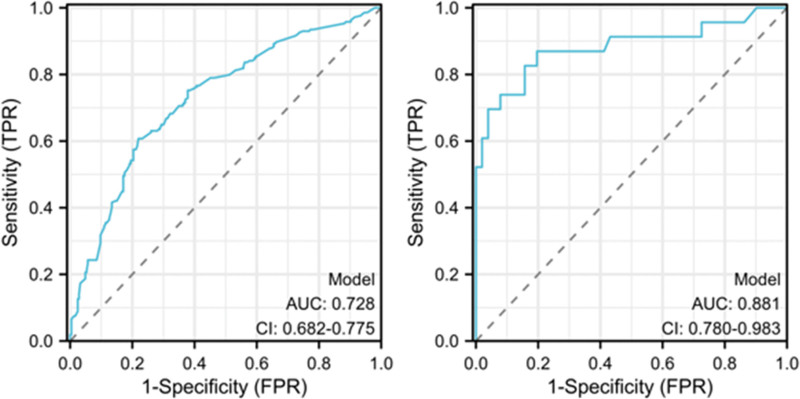
Receiver operating characteristic curve (ROC) validation of hypoproteinemia after TSCI. The y-axis represents the true positive rate of the risk prediction, the x-axis represents the false positive rate of the risk prediction. The thick blue line represents the performance of the nomogram in the training set (A) and validation set (B). TSCI = traumatic spinal cord injury.

**Figure 6.**
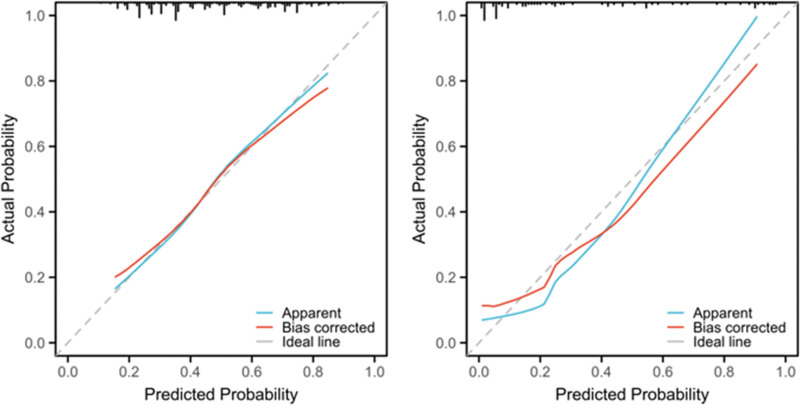
Calibration curves of the predictive hypoproteinemia after TSCI risk nomogram. The y-axis represents actual diagnosed cases of hypoproteinemia after TSCI, the x-axis represents the predicted risk of hypoproteinemia after TSCI. The diagonal dotted line represents a perfect prediction by an ideal model, the solid line represents the performance of the training set (A) and validation set (B), and the results indicating that a closer fit to the diagonal dotted line represents a better prediction. TSCI = traumatic spinal cord injury.

**Figure 7.**
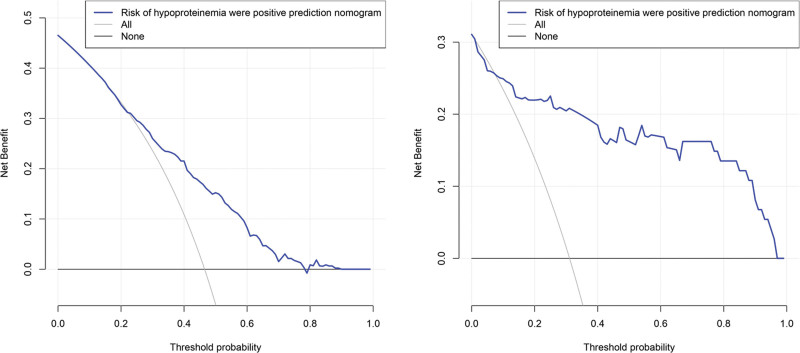
Decision curve analysis (DCA) for the hypoproteinemia after TSCI risk nomogram. The y-axis measures the net benefit. The thick solid line represents the assumption that all patients have no hypoproteinemia after TSCI, the thin solid line represents the assumption that all patients have hypoproteinemia after TSCI, the dotted line represents the risk nomogram. (A) From the training set, (B) from the validation set. TSCI = traumatic spinal cord injury.

The flow chart of the study is shown in Figure 1 and Figure 2 shows a representative radiographic image of a patient with TSCI. Parameters with statistically significant differences in the LASSO regression analysis included male/female sex, age ≥ 60 years, cervical SCI, pneumonia, pleural effusion, urinary tract infection (UTI), hyponatremia, fever, hypotension, and tracheostomy. Binomial deviance (Fig. 3A) and coefficient (Fig. 3B) analyses were performed using LASSO analysis. A novel nomogram for the prediction of hypoproteinemia in patients with TSCI was developed (Fig. 4). The calibration curve of the new prediction model closely resembled the ideal curve, indicating that the model has a favorable predictive ability (Fig. 5). The area under the curve was 0.728 for the training set and 0.881 for the validation set (Fig. 6). The net benefit of the predictive nomogram determined using decision curve analysis met the clinical needs (Fig. 7). The C-index was 0.728.

## 4. Discussion

Hypoproteinemia is an important indicator of adverse outcomes in patients with SCI.^[17]^ Blood tests can be used to detect hypoproteinemia, though patients with TSCI have a long course of disease, and frequent blood sampling results in pain and unnecessary economic burden for patients. Low serum albumin levels have a negative impact on the health of trauma and critically ill patients.^[10]^ Therefore, a tool to predict hypoproteinemia in patients with TSCI, calculate the incidence of hypoproteinemia, and focus on high-risk patients is needed. In this study, a predictive model for the development of hypoproteinemia after TSCI was developed and validated. The results indicated that the model has a satisfactory predictive accuracy.

Albumin is one of the most abundant circulating proteins in blood, accounting for 50% of the plasma proteins. The main physiological functions of albumin include binding to and transporting different medications and substances in plasma, maintaining blood colloid osmotic pressure, and affecting the circulatory system.^[18]^ Albumin also has antioxidant,^[19]^ anticoagulant, and antiplatelet activities.^[20,21]^ Albumin contains a free sulfhydryl group that accounts for 80% of the free sulfhydryl groups in plasma.

Serum albumin levels decrease due to liver damage during the acute phase of inflammation, increased renal excretory proteins, malnutrition, increased catabolism, loss of intestines, severe volume overload, and movement of albumin to the interstitial space, resulting in hypoproteinemia.^[22]^ Hypoproteinemia is defined as a serum albumin level <35 g/L.^[9]^ Patients with severe myocardial infarction or injury, heart failure, stroke, hip fracture, malignant tumors, or kidney disease have decreased serum albumin levels.^[23–25]^ Hypoproteinemia has also been reported in patients with SCI.^[26]^

The long-term mortality of patients with coronary heart disease and hypoalbuminemia is higher than that of patients with normal serum albumin levels.^[27]^ Serum albumin and total cholesterol interact, leading to poor prognosis in patients with coronary artery disease.^[28]^ Hypoalbuminemia may increase the incidence and mortality in hospitalized patients,^[29,30]^ including those with TSCI, in whom persistent hypoalbuminemia is significantly associated with death.^[7,31]^ Reduced serum protein levels associated with hypoalbuminemia and malnutrition are important indicators of mortality in patients with cervical SCI. Early corrective measures for hypoalbuminemia may help reduce the risk of mortality in patients with cervical SCI. Hypoproteinemia is a risk factor in elderly patients with cervical SCI or cervical spine fractures.^[32]^ A multicenter survey of the prognostic factors for respiratory dysfunction in elderly patients with cervical SCI or cervical spine fractures reported that hypoalbuminemia is an independent risk factor for respiratory failure.^[6]^ Screening for hypoproteinemia, identifying high-risk groups, early detection of signs of disease deterioration, and timely and comprehensive treatment will help improve patient prognoses.

Previous studies have confirmed the predictive value of hypoproteinemia in patients with SCI. However, tools for screening and identifying individuals at high risk for hypoproteinemia are lacking. In the current study, a nomogram to predict hypoproteinemia after TSCI was constructed. This study has several advantages. First, the clinical data were from hospitalized patients recruited from 3 hospitals, ensuring the reliability of the established nomogram prediction model. Second, this study implemented external validation, which is necessary to construct a nomogram according to the relevant criteria. Therefore, the developed nomogram is useful for the prediction of hypoproteinemia. Finally, the predictive factors of the nomogram clinical prediction model can be obtained easily. Overall, the model is practical and easy to implement.

However, this study has some limitations. The included patients were mainly from Guangxi, China, which limits the generalizability of the prediction model. Additional factors may need to be considered in other regions or populations. If more comprehensive risk factors are included, verification indicators such as the identification and calibration of the nomogram model may be affected. This poses challenges and opportunities for the universality of the nomogram prediction model developed in this study.

Future studies should include a larger sample size from diverse geographical regions to comprehensively investigate the potential risk factors associated with hypoproteinemia following TSCI and ensure a more reasonable validation of the nomogram clinical prediction model. Furthermore, while this study focused on the prevalence of hypoproteinemia, the findings can serve as a basis for evaluating the incidence of hypoproteinemia after TSCI over 3-, 5-, or 10-year study periods. The development of prediction models of hypoproteinemia after TSCI can be studied in future investigations.

## 5. Conclusion

In summary, hypoproteinemia after TSCI may be predicted using the risk factors of male/female sex, age ≥ 60 years, cervical SCI, pneumonia, pleural effusion, UTI, hyponatremia, fever, hypotension, and tracheostomy. The clinical prediction nomogram developed in this study is straightforward and can facilitate the early detection and diagnosis of hypoproteinemia, leading to improved clinical outcomes. The nomogram can also help achieve effective screening for hypoproteinemia in patients with TSCI.

## Acknowledgments

The authors wish to thank the study participants. The authors also thank their colleagues who coauthored the study discussed here.

## Author contributions

**Conceptualization:** Xiuwei Tan.

**Data curation:** Qian Wei, Xuefeng Lu, Xiaoxi Huang, Deshen He, Xiaozhen Huang, Shiquan Deng, Linting Hu.

**Formal analysis:** Qian Wei, Xuefeng Lu.

**Investigation:** Yanlan Wu.

**Software:** Xiuwei Tan.

**Supervision:** Yiji Su, Fangming Song.

**Validation:** Fengxin Li.

**Writing – original draft:** Xiuwei Tan.

**Writing – review & editing:** Yiji Su, Fangming Song.
